# From Transseptal Puncture to Mitral Commissurotomy: A Zero-Wire-Exchange Approach Using Inoue Balloon

**DOI:** 10.1016/j.jaccas.2026.108027

**Published:** 2026-06-03

**Authors:** Hossameldin Hussein, Ahmed Elsherif, Anwar Hussain, Sagar N. Doshi, Adnan Nadir

**Affiliations:** a Department of Cardiology, Queen Elizabeth University Hospital, Birmingham, UK; b Department of Cardiology, Kasr Al-Ainy Medical School, Cairo University, Cairo, Egypt; c Institute for Cardiovascular Sciences, College of Medical and Dental Sciences, University of Birmingham, Birmingham, UK

**Keywords:** case series, Inoue balloon, mitral valve crossing, percutaneous balloon mitral valvuloplasty, percutaneous mitral commissurotomy, radiofrequency transseptal puncture

## Abstract

Percutaneous mitral commissurotomy (PMC) remains the standard therapy for rheumatic mitral stenosis, although decreasing disease prevalence in developed countries has reduced operator familiarity. Advancing the Inoue balloon across the mitral valve continues to pose a technically demanding step that may extend procedures or result in unsuccessful attempts. We describe 2 cases of severe mitral stenosis successfully treated with PMC using a zero-exchange-wire technique. Transseptal puncture was performed under transesophageal echocardiography guidance using the VersaCross radiofrequency system. The same preshaped 0.035-inch radiofrequency wire was advanced across the mitral valve into the left ventricle, guided by the steerable sheath. The Inoue balloon was then slenderized and delivered over the wire after septal dilatation. Sequential inflations were performed over the wire, achieving optimal results. In summary, we describe a modified over-the-wire technique for PMC that most structuralists are familiar with, using dedicated Inoue balloons, obviating the need for wire exchange, with shorter procedural time and no safety concerns.

## Visual Summary

**Figure:**
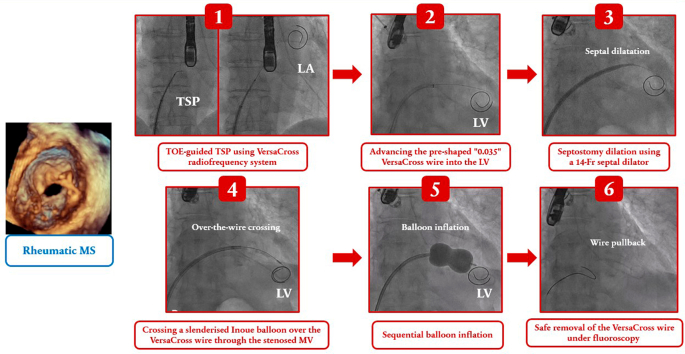

**Visual Summary:**
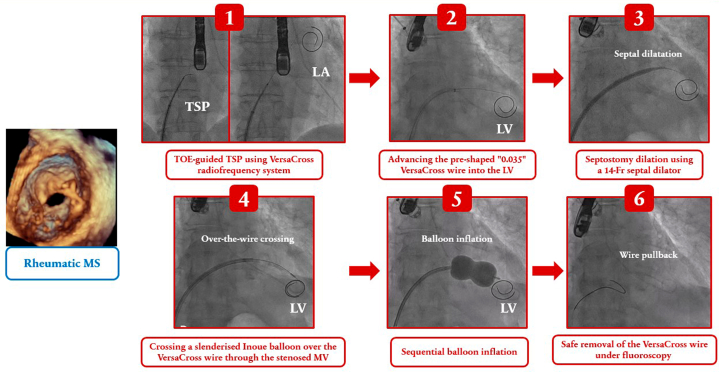
Procedural Steps of Zero-Wire Exchange PMC Using an Inoue Balloon Over the VersaCross Wire LA = left atrium; LV = left ventricle; MS = mitral stenosis; MV = mitral valve; PMC = percutaneous mitral commissurotomy; RF = radiofrequency; TOE = transesophageal echocardiography; TSP = transseptal puncture.

Percutaneous mitral commissurotomy (PMC) is the standard treatment for significant rheumatic mitral stenosis (MS). While the prevalence of rheumatic heart disease is declining in industrialized countries, resulting in a low volume of PMC procedures, it is still high in developing countries, with rates up to 14%.^1^ The recent European Society of Cardiology guidelines recommend that PMC should be restricted to expert operators in specialized centers, particularly in high-income countries, to improve safety and procedural success rate.^2^

**Take-Home Messages**

• Using the 0.035-inch VersaCross radiofrequency wire for both transseptal puncture and mitral valve crossing enables a zero-exchange-wire percutaneous mitral commissurotomy technique, simplifying the procedure, reducing equipment exchanges, and shortening procedural time without compromising safety.
• The smaller preshaped curve and controlled radiofrequency puncture capability of the VersaCross system make it particularly advantageous in rheumatic mitral stenosis with thickened interatrial septum and small left ventricular cavities, offering a reproducible and operator-friendly strategy for challenging Inoue balloon delivery.

The standard approach in PMC involves the exchange of wire after transseptal puncture (TSP). Passing the Inoue balloon (Toray Medical) with the free-floating catheter through the stenosed mitral valve (MV) opening with every inflation is a technically demanding step, resulting in more procedural time, radiation dose, and at times suboptimal or unsuccessful procedure. Some operators in developed countries with very low prevalence have resorted to using aortic valvuloplasty balloon instead of dedicated Inoue balloon given lack of experience. Although other techniques have been described,^3^ they pose certain challenges, for example, the need for multiple sizes of balloons and the use of additional equipment with associated extra costs.

In this case series, we propose a simple and cost-effective technique to perform the entire PMC procedure using a single-wire approach, precluding the need for wire exchange and still using a dedicated Inoue balloon.

## Case Presentation

### Patient 1

A 47-year-old woman was referred for evaluation of severe symptomatic rheumatic MS. She presented with progressive shortness of breath, with a medical history of paroxysmal atrial fibrillation, pulmonary sarcoidosis, pulmonary hypertension, and a previous episode of pulmonary hemorrhage secondary to warfarin therapy. After a multidisciplinary team discussion, PMC was recommended as the preferred therapeutic option, given the rheumatic etiology and coexistence of pulmonary sarcoidosis.

**Figure 1:**
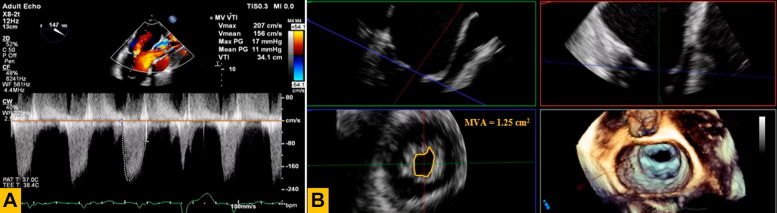
Transesophageal Echocardiography Showing Significant Mitral Stenosis, Patient 1 (A) Mean transmitral gradient of 11 mm Hg. (B) Mitral valve area (MVA) of 1.25 cm^2^ measured by 3-dimensional planimetry.

The procedure was undertaken under general anesthesia. Preprocedural transesophageal echocardiography (TEE) confirmed hemodynamically significant rheumatic MS, with a mean transmitral gradient of 11 mm Hg and a mitral valve area (MVA) of 1.25 cm^2^ according to 3-dimensional planimetry (Figure 1). Mitral regurgitation (MR) was mild, and the Wilkins score was favorable for the procedure.

**Figure 2:**
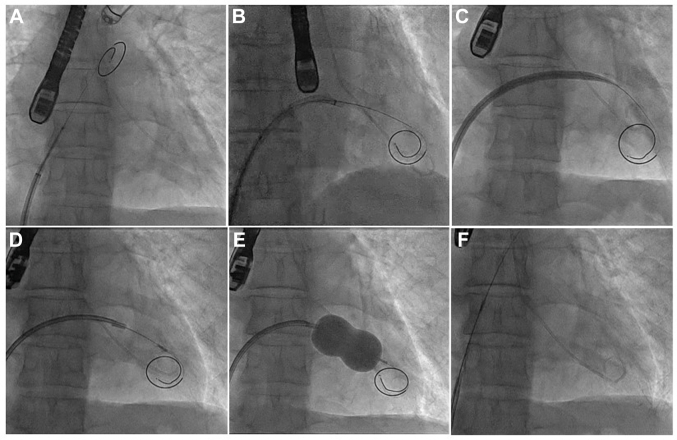
Procedural Steps, Patient 1 (A) Transseptal puncture using an 8.5-F VersaCross system with advancement of the preshaped VersaCross wire into the left atrium. (B) The VersaCross wire was further advanced into the left ventricle, directed by the VersaCross steerable sheath. (C) Septostomy is dilated using a 14-F septal dilator that is advanced over the VersaCross wire. (D) Delivery of a slenderized 26-mm Inoue balloon via the modified over-the-wire technique to cross the diseased mitral valve. (E) Sequential balloon inflations to a final diameter of 26 mm. (F) Direct withdrawal of the balloon and guidewire from the left ventricle under fluoroscopic guidance.

Primary venous access was obtained via the right femoral vein using ultrasound guidance and a 14-F sheath deployed after a single ProGlide suture. Arterial access was achieved through the right radial artery, and a 6-F pigtail catheter was positioned in the noncoronary aortic cusp. A TSP was performed using an 8.5-F steerable VersaCross system (Baylis Medical) (Figure 2A, Video 1). Through the VersaCross sheath, the preshaped 0.035-inch guidewire was advanced directly into the left ventricle (LV) with no wires exchanged (Figure 2B, Video 2). The arterial pigtail catheter was advanced into the LV for hemodynamic measurements. The interatrial septostomy was then dilated using a 14-F kit dilator over the VersaCross wire (Figure 2C, Video 3).

**Figure 3:**
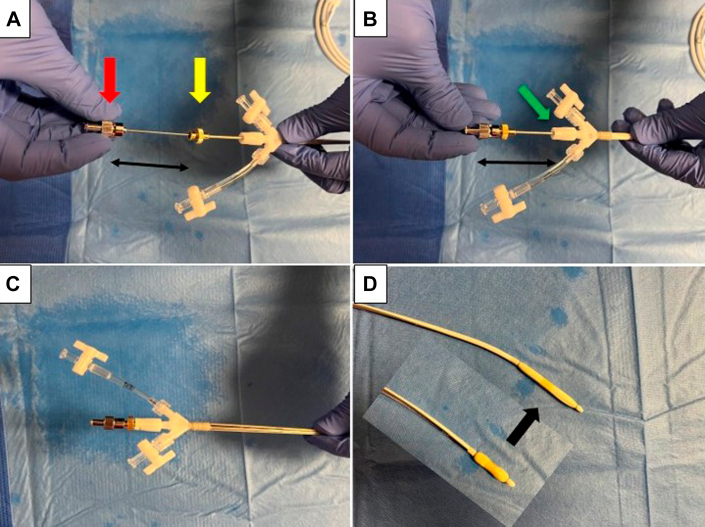
Standard Inoue Balloon Preparation (A to D) Slenderized position is obtained through locking the stretching tube (red arrow) to the inner lumen tube (yellow arrow), then locking the inner tube to the hub (green arrow) of the balloon catheter. The slenderized balloon is then advanced over the Inoue guidewire across the septostomy. Once in the left atrium, the above steps are reversed, and balloon is floated across the mitral valve with the help of a shapeable stylet.

**Figure 4:**
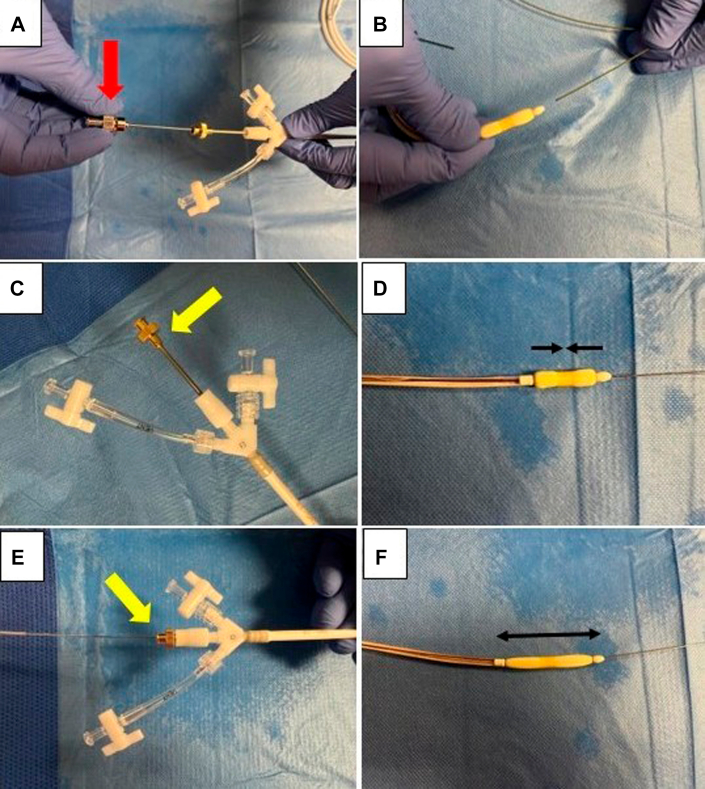
Modification of Inoue Balloon Preparation (A) The stretching silver tube (not compatible with 0.035-inch VersaCross wire) is removed (red arrow). (B) The front end of the Inoue balloon is loaded over the back end of the VersaCross guidewire (after transseptal puncture). (C and D) Resting unslenderized position. (E and F) Slenderized position is obtained outside the body through locking the inner tube to the hub of the balloon catheter back end (yellow arrow), causing balloon elongation while on the wire. The balloon is then advanced smoothly over the VersaCross guidewire across the septostomy. Once correctly positioned in the left atrium, the balloon is unslenderized again by retracting the inner tube, then advanced to the left ventricular cavity and directly inflated over the VersaCross guidewire. No need for Inoue guidewire or the shapeable stylet.

The Inoue balloon was prepared in standard fashion except the insertion of a slenderizing metal tube (Figures 3 and 4). The balloon was slenderized outside, after loading over the preshaped LV guidewire, and was advanced smoothly across the septostomy into the MV (Figure 2D, Video 4). Once correctly positioned, the balloon was unslenderized, and 3 sequential inflations were performed, starting at 22 mm and increasing to a final diameter of 26 mm (Figure 2E, Video 5). Since there was no slenderizing metal tube, the catheter lumen was adequate to continuously monitor pressure through the balloon tip; hence, it was possible to measure tranmitral gradient after each inflation without losing LV wire position.

**Figure 5:**
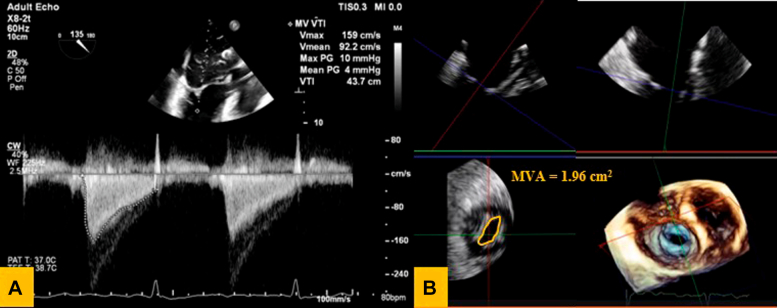
Postinterventional Transesophageal Echocardiography, Patient 1 (A) A drop in the mean transmitral gradient to 4 mm Hg. (B) An increase in mitral valve area (MVA) to 1.96 cm^2^ measured by 3-dimensional planimetry.

Stepwise TEE assessment demonstrated an improvement in MVA to 1.96 cm^2^, and the mean transmitral gradient dropped to 4 mm Hg, with no change in the degree of MR (Figure 5). The Inoue balloon and guidewire were pulled directly from the LV under fluoroscopy (Figure 2F, Video 6). The femoral access site was closed using the predeployed single ProGlide suture, achieving hemostasis.

### Patient 2

An 82-year-old woman with a history of rheumatic MS was referred with worsening breathlessness. She had a previous history of repeated MV interventions, including surgical mitral commissurotomy 50 years ago as well as 2 PMC interventions 10 years and 2 years ago. The last PMC procedure was performed using a 26-mm Inoue balloon, which improved the MVA from 1.4 to 1.8 cm^2^, with reduction in the mean gradient from 9 to 5 mm Hg. Other comorbidities included myelodysplasia and chronic anemia.

**Figure 6:**
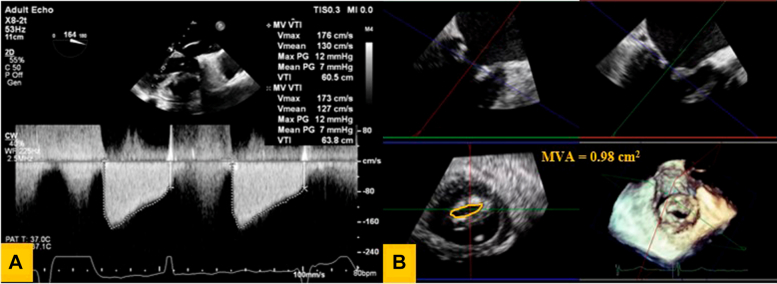
Transesophageal Echocardiography Demonstrating Severe Mitral Stenosis, Patient 2 (A) Mean transmitral gradient of 7 mm Hg. (B) Mitral valve area (MVA) of 0.98 cm^2^ measured by 3-dimensional planimetry.

TTW demonstrated an MVA of 0.98 cm^2^ by planimetry and a mean gradient of 7 mm Hg (Figure 6). Biventricular size and systolic function were preserved. Given the patient's age and frailty, her case was rediscussed in the multidisciplinary team meeting, and it was agreed that she remained a poor surgical candidate and would benefit from another PMC, with the option to use a larger balloon size (28 mm).

**Figure 7:**
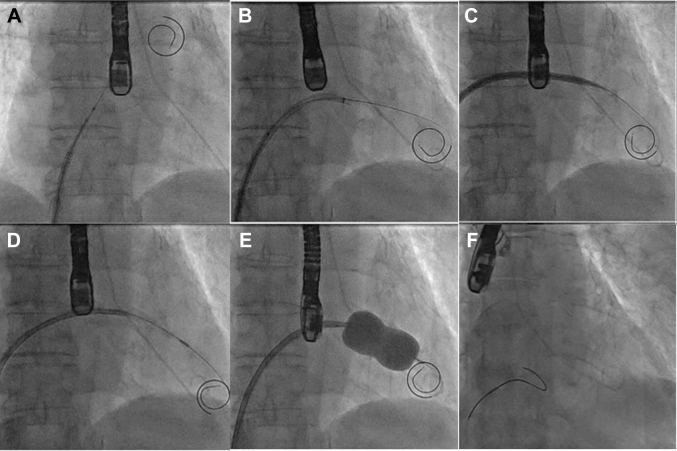
Procedural Steps, Patient 2 (A) Transseptal puncture performed with an 8.5-F VersaCross system, with advancement of the preshaped guidewire into the left atrium. (B) Further advancement of the VersaCross wire into the left ventricle, guided by the steerable VersaCross sheath. (C) Septostomy dilation using a 14-F septal dilator. (D) Delivery of a slenderized 28-mm Inoue balloon across the diseased mitral valve using a modified over-the-wire technique. (E) Three sequential balloon inflations. (F) Direct withdrawal of the balloon and guidewire from the left ventricle.

**Figure 8:**
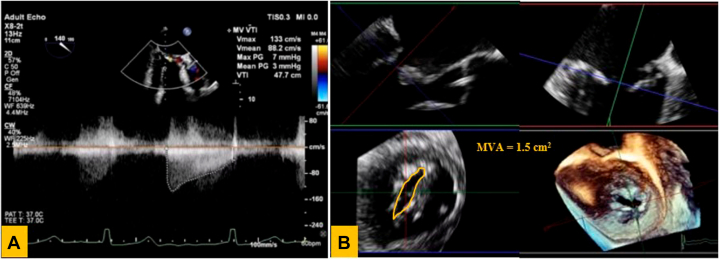
Postinterventional Transesophageal Echocardiography, Patient 2 (A) Reduction in the mean transmitral gradient to 3 mm Hg. (B) Increase in mitral valve area (MVA) to 1.5 cm^2^ as assessed by 3-dimensional planimetry.

The patient underwent PMC under general anesthesia with TEE guidance. Venous access was obtained via the right femoral vein and secured with a ProGlide suture, and a 14-F sheath was inserted. Right radial arterial access was used for hemodynamic monitoring. A TSP was performed using an 8.5-F steerable VersaCross sheath (Figure 7A), and a preshaped 0.035-inch guidewire was steered into the LV to provide stable access to the MV (Figure 7B). The interatrial septum (IAS) was then dilated using a 14-F septal dilator over the VersaCross guidewire (Figure 7C). The modified over-the-wire technique was used to deliver a prepared 28-mm Inoue balloon, slenderized over the wire and advanced across the septostomy into the MV (Figure 7D). After positioning, the balloon was unslenderized, and 3 sequential inflations were carried out (Figure 7E), achieving satisfactory results with a reduction in the mean gradient to 3 mm Hg and increase in MVA to 1.5 cm^2^ on 3-dimensional planimetry assessment (Figure 8). MR remained unchanged. The Inoue balloon and LV guidewire were safely pulled and removed under fluoroscopy (Figure 7F). Hemostasis of the venous access was achieved using the predeployed ProGlide suture.

## Discussion

PMC is the gold-standard treatment of rheumatic MS, with established evidence of superiority to MV surgery in cases of favorable anatomy. Owing to the low prevalence of rheumatic heart disease, it is not widely performed in many countries, resulting in fewer experienced operators and a steeper learning curve. Over time, the technique of PMC has undergone some modifications, with the introduction of new equipment to facilitate TSP and overcome the technical challenges of the Inoue balloon crossing through the stenosed MV.^4^

### Technical challenges of the standard Inoue technique

In the standard Inoue technique, puncturing the IAS is performed using a Brockenbrough needle through an 8-F Mullins introducer sheath. A 0.025-inch spring guidewire is then introduced into the left atrium, and the Mullins sheath is exchanged with a 14-F septal dilator. After septal dilation, the Inoue balloon catheter is introduced into the left atrium over the coiled Inoue spring guidewire.

PMC using the classic Inoue technique can be technically challenging, especially in patients with difficult anatomies. Failure of the procedure is mainly attributed to difficult TSP or inability to cross the MV. Critically severe MS with an MVA of <0.5 cm^2^ or a heavily diseased subvalvular apparatus usually interfere with the standard technique. A thick or bulging IAS may add to procedural complexity, causing a shift in the ideal TSP location leftward or downward.

### Alternative strategies to the standard technique

In case of failure to cross the MV, changing the shape of the J-shaped 0.038-inch guiding stylet may be initially attempted to direct the balloon toward the MV orifice. Over-the-wire entry into the LV is an alternative strategy that increases the success rate in these complex anatomical scenarios.^5^ The risk of frequent ventricular ectopics or sustained ventricular arrhythmia is low, with no reported hemodynamic compromise, LV perforation, cardiac tamponade, or procedural mortality. Nevertheless, this approach involves multiple steps with repeated wire exchanges, resulting in higher costs and increased operator learning curve.

Momen et al^1^ proposed an effective approach to overcome challenging MV crossing, in which the balloon is removed, keeping the spring guidewire in the left atrium. Then, the Mullins sheath is introduced over the wire, and the tip of the wire is directed to the LV cavity during diastole. This is followed by sheath removal and balloon introduction over the wire into the LV. In the same study, modifying the fluoroscopy from posteroanterior to right anterior oblique view increased the overall rate of MV crossing.^1^

An arteriovenous loop technique has also been described in PMC procedures. An arteriovenous rail may be created using a 0.035-inch Terumo wire to assist PMC balloon tracking across the MV.^6^ A multipurpose 6-F catheter is introduced via Mullins long sheath into the LV, inserting an exchange-length 0.035-inch Terumo wire into the LV and across the aortic valve into the descending aorta. The Terumo wire is then snared in the descending aorta with a snare kit supported by a JR 6-F diagnostic catheter. In a hugely dilated LA cavity, a double loop of the Inoue balloon may be performed via counterclockwise rotation to facilitate crossing.^7^

### Preshaped 0.035-inch Safari stiff wire

Recently, Gupta et al^8^ described a new technique that involved crossing a preshaped 0.035-inch Safari XS wire (Boston Scientific) into the LV, on which the Inoue balloon is slenderized and guided through the MV. In this approach however, the VersaCross wire is exchanged with the stiff wire, which is then directed using a steerable Agilis sheath (Abbott Vascular). The pivotal difference in our described technique is obviating the need for these extra steps or the use of additional equipment with associated costs.

### Advantages of our approach using the VersaCross system

The TSP technique has evolved over the past several years. It is conventionally performed with solid-tipped needles or preshaped nitinol needle-tipped wires. Radiofrequency (RF) energy needles were then introduced, with reported higher success rates, shorter duration, and less risk of perforation or tamponade.^9^ RF catheters are particularly useful in thickened septal anatomy, which is common in rheumatic MS.

The VersaCross system is a relatively new RF platform designed to perform TSP. It consists of a 180-cm 0.035-inch pigtail wire that is connected to an external RF generator, a reinforced shapeable dilator, and a steerable transseptal sheath. Compared to RF needles, the use of RF wires for TSP has been associated with shorter time and fewer equipment exchanges, with similar safety.^10^

In our described technique, both TSP and LV crossing are performed using the same VersaCross wire. The external energy used for TSP was 60 W for 1 second. With flexion of the VersaCross steerable sheath, the wire is directed into the LV. An Inoue balloon is then prepared in the usual way except for the metal slenderizing tube, which is not inserted. The VersaCross wire has a smaller curve compared to extra-stiff wires (curve is 2.4 cm^2^ vs 2.9-3.2 cm for stiff wires), rendering it more suitable and safer for small LV cavities in patients with MS.

### Technical tips and pitfalls

1. Although TSP can be guided by fluoroscopy only, with the use of transthoracic echocardiography for assessment of the final outcomes, we used standard TEE guidance under general anesthesia in all our cases.
2. The VersaCross steerable sheath can be flexed to facilitate wire crossing, but if needed a pigtail can be mounted on the RF wire in case of difficulty and then advanced without need to exchange (Video 2).
3. The Inoue balloon can be introduced directly without a sheath. Current practice involves balloon advancement through a 14-F sheath, without risk of balloon damage. A 16-F sheath is sometimes used to facilitate removal of the deflated balloon out. In all our cases, we used the 14-F sheath, with no encountered challenges or risks.
4. A pigtail catheter can be used for advancing or removal of an extra-stiff wire into the LV. However, both direct advancing and removal of the VersaCross wire through the MV were safe, given less stiffness and the small curve.
5. If the VersaCross wire is dislodged from the LV, the standard technique for PMC can still be used without need to exchange the wire.
6. Some manipulation and rotation of the balloon during advancement through the septum over the wire may be required (Video 4).

## Conclusions

In PMC, the preshaped 0.035-inch VersaCross RF guidewire can be used for over-the-wire advancement of the Inoue balloon into the LV. This strategy eliminates the need for wire exchange, streamlines the entire procedure, shortens procedural time, and demonstrates consistent feasibility and safety in different clinical scenarios.

## Funding Support and Author Disclosures

The authors have reported that they have no relationships relevant to the contents of this paper to disclose.

## Appendix

Video 1 Transseptal Puncture Using the VersaCross Radiofrequency System

Video 2 Advancing the Preshaped 0.035-inch VersaCross Wire Into the Left Ventricular Cavity

Video 3 Septostomy Dilation Using a 14-F Septal Dilator

Video 4 Crossing a Slenderized Inoue Balloon Over the VersaCross Wire Through the Septum Then Mitral Valve

Video 5 Inoue Balloon Inflation

Video 6 Safe Removal of the VersaCross Wire Under Fluoroscopy
